# High throughput evaluation of macrocyclization strategies for conformer stabilization

**DOI:** 10.1038/s41598-018-24766-5

**Published:** 2018-04-26

**Authors:** Dan Sindhikara, Ken Borrelli

**Affiliations:** Schrodinger, Inc. Department of Life Sciences, New York, NY 10036 USA

## Abstract

While macrocyclization of a linear compound to stabilize a known bioactive conformation can be a useful strategy to increase binding potency, the difficulty of macrocycle synthesis can limit the throughput of such strategies. Thus computational techniques may offer the higher throughput required to screen large numbers of compounds. Here we introduce a method for evaluating the propensity of a macrocyclic compound to adopt a conformation similar that of a known active linear compound in the binding site. This method can be used as a fast screening tool for prioritizing macrocycles by leveraging the assumption that the propensity for the known bioactive substructural conformation relates to the affinity. While this method cannot to identify new interactions not present in the known linear compound, it could quickly differentiate compounds where the three dimensional geometries imposed by the macrocyclization prevent adoption of conformations with the same contacts as the linear compound in their conserved region. Here we report the implementation of this method using an RMSD-based structural descriptor and a Boltzmann-weighted propensity calculation and apply it retrospectively to three macrocycle linker optimization design projects. We found the method performs well in terms of prioritizing more potent compounds.

## Introduction

Macrocycles are an important and pervasive class of molecule for drug design^1^. Though definitions vary, they are typically described as molecules with ring sizes of at least 8, 10, or 12 atoms^2,3^. They span a molecular weight range typically larger than small molecules and smaller than biological therapeutics^4^. Though some design projects begin with a macrocyclic native ligand, often a cyclization will be performed to introduce a conformational restriction to an otherwise linear molecule^5–7^. Regardless, the cyclization topology can drastically affect the conformational propensity of the molecule and alter the affinity of the molecule for its target receptor. Understanding this conformational effect on bioactivity is essential to macrocycle design.

*In silico* sampling of macrocycles is particularly difficult due to the conformational restriction imposed by the cyclization. Several methods have been developed to overcome this sampling challenge including distance-geometry-based^8^, low-mode based^9^, normal-mode-based^10^, inverse kinematics-based^11^, and loop-sampling-based^12^ conformational searches.

Previous work has created workflows to screen macrocycles by combining these sampling methods with scoring functions such as molecular mechanics simulated annealing combined with quantum mechanical strain scoring^13^, inverse kinematics with ROSETTA^14^, implicit solvent/molecular mechanics scoring^15^, and normal-mode-based sampling with molecular mechanics scoring^16^. In this work, we implement an integrated, high-throughput method utilizing a loop-sampling-based macrocycle conformational sampling protocol along with a simple molecular mechanics strain-based scoring function.

The ability to access a given binding conformation is one of the many factors that determine the binding affinity of a specific ligand for a specific protein. When the binding mode is assumed to be both known and constant, this can be treated as a necessary, but not sufficient, condition for high affinity binding. While this applies to all molecules, it is especially important for macrocyclic ligands due to the restriction of conformational space caused by the cyclization. When comparing different potential cyclizations of a linear molecule where the linker is not interacting with the protein, this could even become the primary factor affecting the relative binding energy of a series of compounds. In these cases, determining the relative ability of a various cyclized versions of the same linear molecule to adopt a known binding model may be able to serve as a proxy for the relative binding affinity of those compounds. In this work we will show that the propensity of a macrocycle to adopt a specific binding mode, also termed the bioactive conformation of that macrocycle, can explain the differences in binding affinity for sets of congeneric macrocyclic molecules when the only differences between those macrocycles are in the linker region and that linker region is not making any contacts with the protein.

Previous work has shown the ability of Prime Macrocycle Sampling to efficiently sample the conformational space of macrocycles^12^ and here we combine it with the OPLS3 force^17^ field to evaluate whether molecular mechanics approaches, which are significantly computationally cheaper than quantum approaches, are adequate to determine the relative strain energies of these compounds. Unlike the inverse kinematic approach previously reported, this method would be expected to be work with macrocycles that did not have additional crosslinks to reduce the conformational space to be sampled. The approach presented here is similar to that used by McCoul *et al*. to design macrocyclic BCL6 inhibitors^16^, however here we demonstrate an integrated workflow and validate it on diverse test sets.

## Methods

### Conformer generation

Although generation of conformational ensembles of macrocycles is difficult, many methods have been developed to overcome the particular difficulties of sampling introduced by macrocyclization. Here we use the Prime Macrocycle Conformational Sampling algorithm (Prime-MCS) which can quickly produce accurate, diverse macrocycle conformations^12^. Default calculation parameters were used including unbiased PrimeMCS sampling and OPLS3.0 force field parameters^17^ in vacuum. This assumes that unbiased default sampling is sufficient to cover representative conformational space and that the OPLS3 force field can sufficiently represent the compound. It is possible that, depending on the design context, especially the macrocycle topological complexity, that these assumptions will be broken and the sampling protocol should be modified. Some such modifications are discussed in the Discussion section.

### Ensemble scoring

#### Conformer structural metric

To properly score the conformational ensemble, a metric for the similarity to the bioactive substructure is required. For many projects, the conserved substructure is obvious since the linker modifications leave the original acyclic molecule largely unchanged. Here, we apply the *maximum common substructure* (MCS) algorithm, as implemented in Canvas software^18^, to the designed macrocycles and the bioactive linear reference to determine the conserved region. Though this approach may not be optimal for use in prospective projects, the MCS is useful under the assumption that the topological differences between the macrocyclic designs and the reference are purely in the cyclization linker, and thus the bioactive conformation of the remaining substructure is intended to be conserved. We take the RMSD of the heavy atoms in the conserved region, *RMSD*_*cons*_ as a simple metric for similarity to the bioactive substructure under the assumption that, for the sets of macrocycles we will be considering, the ability for these atoms to adopt a conserved binding mode is the primary determinant of binding affinity. Alternate structural metrics are discussed below in the *Discussion* section. When measuring the *RMSD*_*cons*_, we account for symmetry by generating a SMARTS pattern from the MCS atoms in the reference ligand and choosing the minimum RMSD for all matches to the SMARTS patterns in the test compounds. Here, the MCS atoms were calculated using Schrodinger’s canvas_MCS utilitity *($SCHRODINGER/utilities/canvas_MCS)* using default settings in Schrodinger release 2017-3^18^. This MCS technique distinguishes atoms by atomic number, bond order, and aromaticity.

#### Ensemble weighting and the proxy metric for affinity

If the conformational ensemble were complete and representative, ensemble weighting would not be necessary. However for biased or clustered methods, reweighting is necessary to recover the correct ensemble. The conformer generation algorithm here, Prime-MCS, uses structure-based cluster output. Such clustered structures may be weighted to the canonical ensemble using simple Boltzmann-weighting (assuming these clusters are evenly distributed in conformational state space). That is, that each conformer is weighted according to its energy (*P*_*i*_ = *exp(−E*_*i*_*/kT)/Z*). This approach has been used, for example, for finite-reservoir sampling methods^19–21^.

After weighting the ensemble, we can calculate the probability distribution of observables. We assume that the affinity is a function of this conformational distribution. Here, we simply use the expectation value of the metric, $\langle RMS{D}_{cons}\rangle ={\sum }_{i}{P}_{i}RMS{D}_{cons,i}$ as the proxy for affinity. The expectation value is useful if the affinity gets better the closer the compound gets to the bioactive structure. Alternate proxy metrics are discussed later in the *Discussion* section.

### Dataset preparation

**Figure 1:**
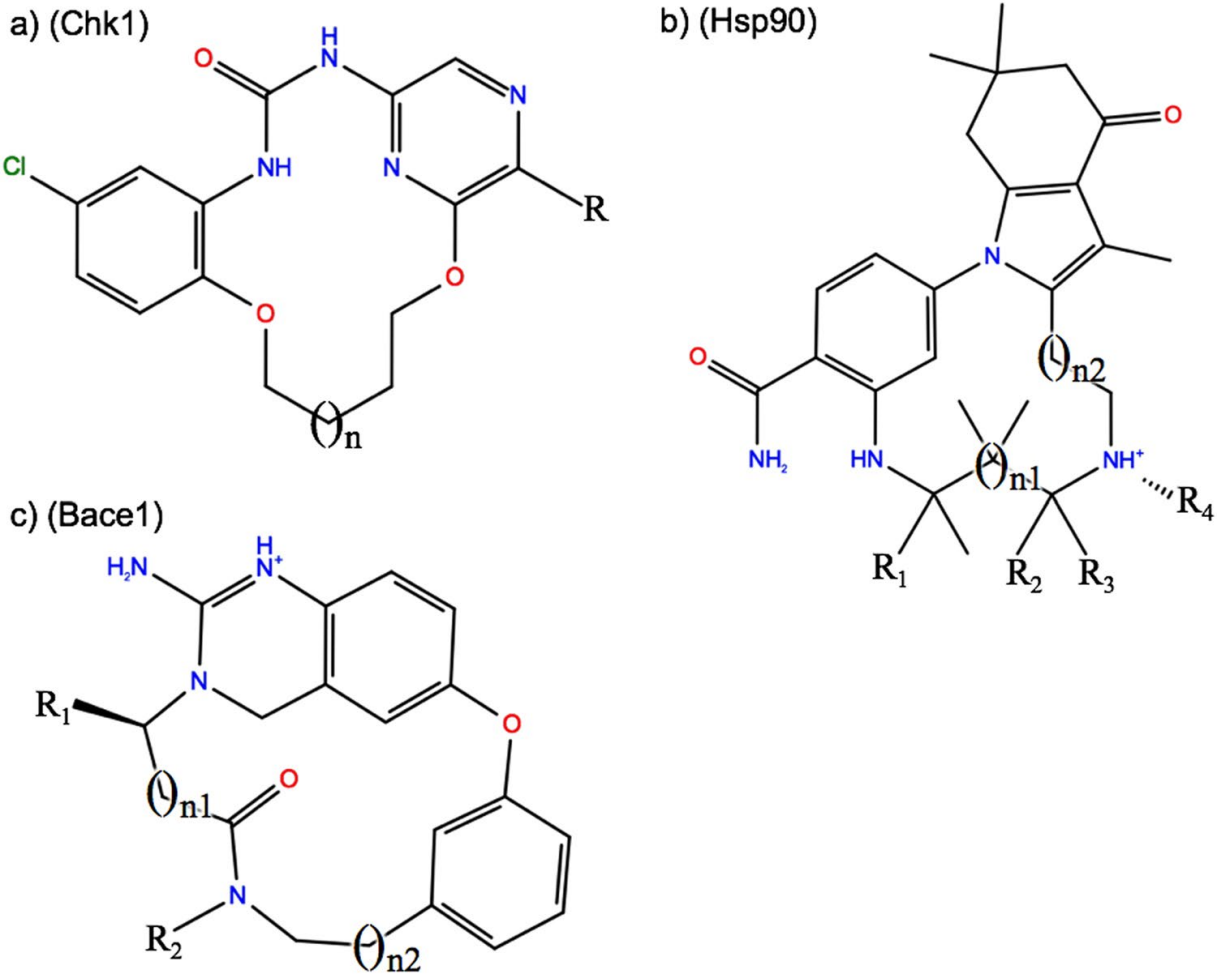
(**a**–**c**) Markush representations of macrocycle candidate compounds.

For this study we use systems from macrocycle design projects taken from the literature. In a related paper submitted recently, seven macrocyclization projects were curated to evaluate free energy perturbation (FEP) technology on affinity calculations for macrocycles^22^. For that article, papers were chosen describing projects which contained macrocyclizations (conversion of linear compounds to macrocyclic), modification of macrocycle linker sizes, or both. Projects were only used if there were no apparent issues with the data that would complicate an evaluation by atom-scale biophysical modeling such as significant missing structural data and insufficient affinity data. Here we select from those seven projects the only three where there were at least two macrocycles within the set. Those systems were Chk1 (PDBID 2E9P), Bace-1 (PDBID 2Q15)^23^, and Hsp90 (PDBID 3RKZ)^24^, see Table 1 and Figure 1. Though protein structures are available, they were not used for any part of these calculations except to confirm that the various modifications to the linker are on the solvent exposed side of the ligand and therefore less likely to be involved in specific protein-ligand interactions that could affect the binding affinities of these molecules.

**Table 1:** Markush representations of enumerated macrocycles with associated substitutions and experimental affinities (*ΔG*_*bind*_ in kcal/mol).

Chk1							
**Cpd**	**R**				**n**		**Exp. dG**_**bind**_
1	H				2		−10.88
2	CN				1		−11.18
3	CN				2		−11.09
4	CN				3		−10.27
**Hsp90**							
**Cpd**	**R**_**1**_	**R**_**2**_	**R**_**3**_	**R**_**4**_	**n1**	**n2**	**Exp. dG**_**bind**_
5					0	1	−6.67
6		CH_3_	CH_3_		0	2	−9.32
7					1	2	−8.89
8	CH_3_			CH_3_	0	2	−9.28
9			-CH_2_-CH_2_-CH_2_-	-CH_2_-CH_2_-CH_2_-	0	2	−8.45
10	CH_3_	CH_3_			0	2	−13.14
11	CH_3_	CH_3_			0	2	−12.74
**Bace1**							
**Cpd**	**R1**	**R2**			**n1**	**n2**	**Exp. dG**_**bind**_
12	H	Cyclohexyl			1	2	−9.82
13	Cyclohexyl	Cyclohexyl			2	1	−11.29
14	Cyclohexyl	Cyclohexyl			2	2	−10.21
15	Cyclohexyl				2	1	−10.57

For the calculations here, the experimental structural data for the macrocyclic structures were removed by converting the structures to SMILES then back to 3D. These 3D structures were then inspected to ensure correct stereoisomer states as well as trans amide bonds. These, as well as the linear reference structure, were prepared using LigPrep^25^.

## Results

### Determining conserved bioactive substructure

**Figure 2:**
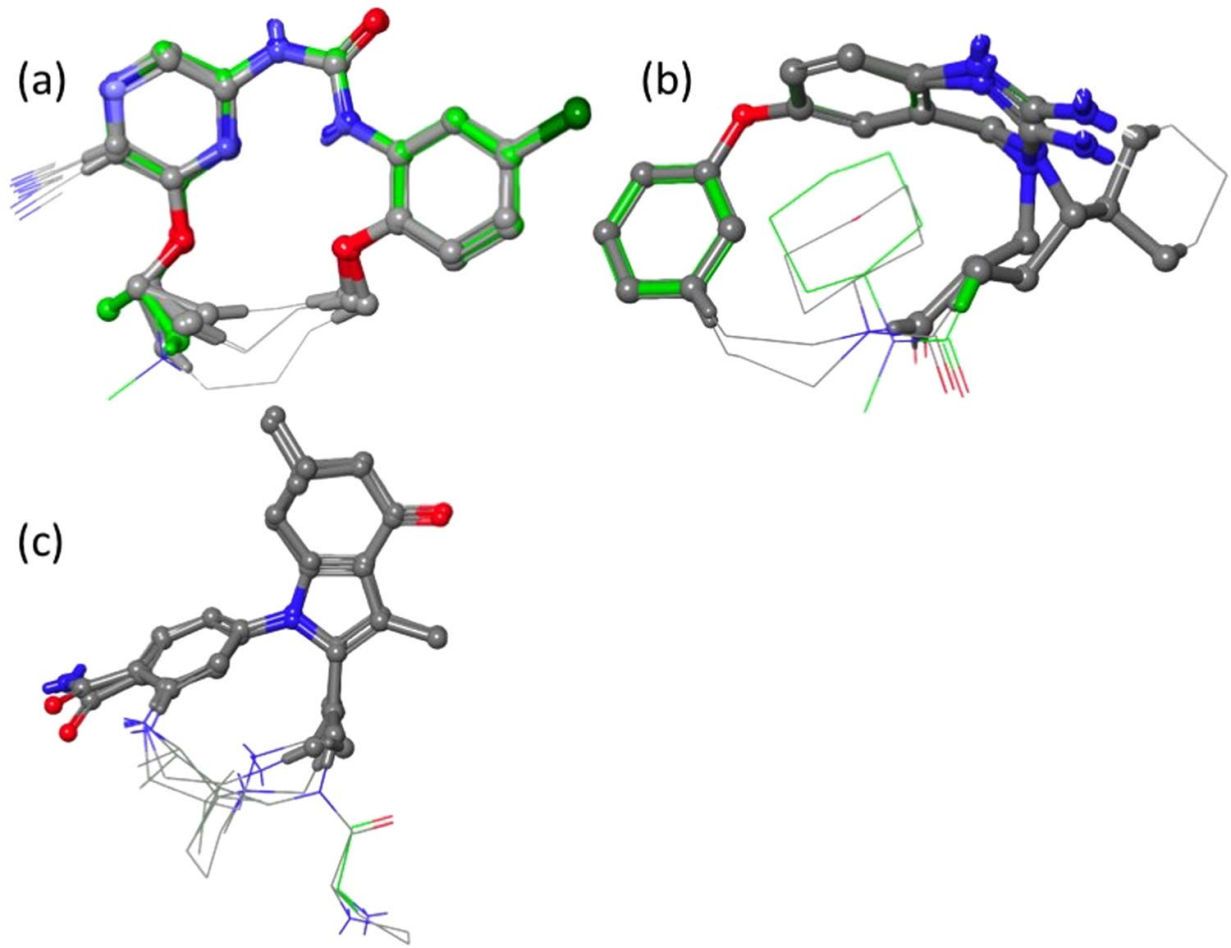
(**a**–**c**) Macrocyclic ligands restraint and superimposed to their bioactive linear references. Reference ligands shown with green carbons. MCS atoms shown as balls and sticks. For Chk1, Bace-1 and HSP90, the MCS SMARTS patterns were, c1c(Cl)ccc(OC)c1NC(=O)Nc2cncc(n2)OCC, CCCN(C1)C(N)=[N+]c(c12)ccc(c2)Oc3ccccc3, and c1cc(C(N)=O)ccc1n(c(c2C)CC)c(c23)CC(C)(C)CC3=O respectively.

Figure 2a–c shows the macrocyclic designs restrained to and superimposed onto their crystal bioactive references with conserved atoms highlighted. As is shown in this figure, the conserved atoms comprise the majority of the heavy atoms. While the MCS approach to determining the conserved atoms may be a reasonable naive guess, the MCS atoms do not necessarily represent the most important atoms in terms of bioactive structure. For example in Chk1, the conserved includes atoms span across the aryl ethers. It could be conjectured that these ethers serve only as structural atoms and thus the representative bioactive substructure should be curtailed at the aromatic rings. Additionally, the MCS algorithm is unaware of conformation, so for Chk1, there is an off-linker carbon included in the conserved region. Since the *RMSD*_*cons*_ calculations use the minimum RMSD across all degenerate matches, this is partially accounted for. But the calculation could still incorrectly favor macrocycles which prefer the wrong conformation of this MCS atom. Such issues would not occur if conserved atoms were manually selected by the macrocycle designer.

### RMSD vs energy plots

**Figure 3:**
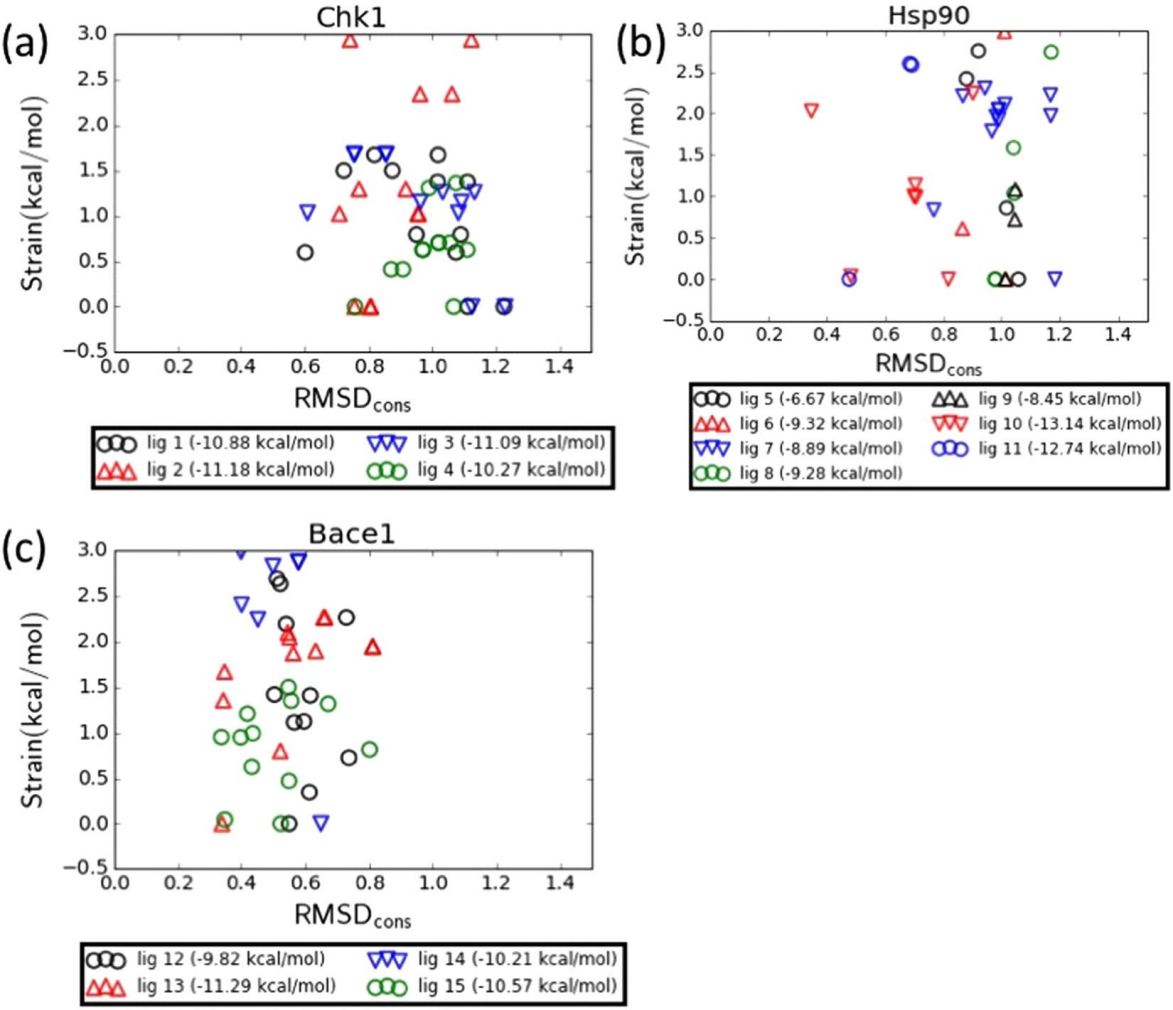
(**a**–**c**) Relative energy of conformation (strain) vs RMSD in the MCS region relative to the reference for Chk1, Hsp90 and Bace1. Each conformation represents one point. Only the 12 lowest strain conformations for each compound are shown. In parentheses the legends indicate the experimental binding affinity.

Figure 3 shows the relative energy of each output conformation (strain) vs the *RMSD*_*MCS*_. The nature of the conformation generation algorithm generates an ensemble of conformers spanning both *RMSD*_*cons*_ and strain space. Different compounds will have different conformational landscapes, and, importantly, may or may not be able to adopt low strain, low *RMSD*_*cons*_ conformations (bottom left corner of the graphs). As can be seen, the compounds with better affinity (lower *ΔG*_*bind*_) tend to better occupy this space. The only clear exception is Chk1 ligand 3 which despite having binding affinity only 0.09 kcal/mol greater (within experimental error) of compound 5, does not contain any conformations in this space. This is likely due to the conserved atom selection (discussed in the Discussion section).

### Expected RMSD vs affinity

**Figure 4:**
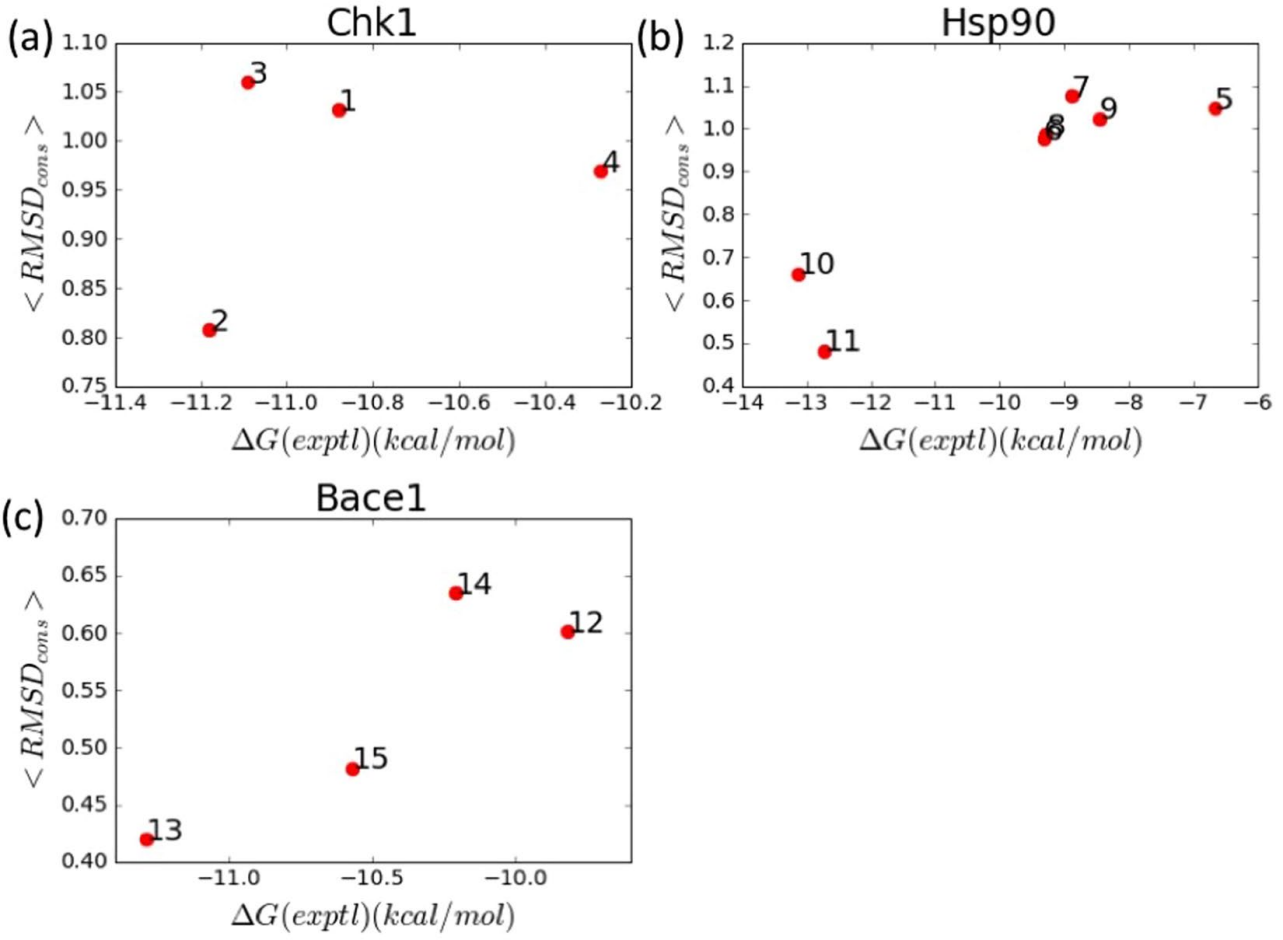
(**a**–**c**) Proxy affinity metric, <*RMSD*_*cons*_> vs experimentally measured affinity for Chk1, Hsp90 and Bace1. Here, no effort was made to optimize the conserved region (see Discussion).

Though the *RMSD*_*cons*_ vs energy plots can be useful for analyzing the conformational space of these candidate compounds, for quantitative comparison, a single metric as a function of these data is necessary. Figure 4a–c shows the Boltzmann-weighted expectation value of the *RMSD*_*cons*_ among the output ensemble. Here, we expect the lower expected RMSD to have the lower *ΔG*_*bind*_. For Hsp90 and Bace-1, the more potent compounds are clearly separated from the rest. For Chk1 despite correctly ranking the most potent compound (ligand 2) as having the lowest expected RMSD, the intermediate compounds do not correlate well (especially ligand 3 as noted in the previous section). The Pearson correlation coefficients were 0.21, 0.90, and 0.90 for Chk1, Hsp90, and Bace-1 respectively, giving a weighted average R^2^ of 0.60. But for Chk1 and Bace1, which had only four data points each and dynamic ranges less than 1.5 k cal/mol, these correlations were not significant (p-values were 0.78 and 0.10 respectively). The Hsp90 set rather, containing seven data points across 7 kcal/mol, had a p-value of 0.005.

### Calculation time

The vast majority of the calculation time for this algorithm is in the sampling since the *RMSD*_*cons*_ and <*RMSD*_*cons*_> algorithm takes only a few seconds per compound on a CPU. The PrimeMCS sampling algorithm is parallelized over 10 threads per compound. For all the sets here, the median parallel calculation time on commodity CPU workstations was 88 seconds per compound, or about 12 minutes per compound on a single CPU. Thus, each CPU could profile over 120 compounds in a single day, giving designers with even modest resources the ability to screen thousands of compounds per day for similarly-sized macrocycles. As noted in the PrimeMCS reference^12^, speed will vary depending on the conformational complexity of the macrocycle and additional sampling or appropriate restraints may increase the accuracy of the method.

## Discussion

### Limitations of the hypothesis

As mentioned previously, there are strong assumptions in the hypothesis underlying this method. It assumes that higher propensity for the purported bioactive substructural conformation leads to higher affinity. First, this assumption excludes possibility of interactions of the nonconserved regions with the protein and any binding solvation effects. However, since many linker designs are in solvent exposed regions, such effects may be small depending on the choice and size of linker. Secondly, the assumption excludes the possibility that the known active structure may not adopt the ideal geometry, that alternate conformations may also imbue activity, or even that some ligand flexibility is required to maintain interactions in a flexible active site. The ability to dismiss these possibilities must be determined on a per-project basis. Due to these limitations, the method may not be applicable to all macrocycle design projects or may only be applicable in portions of the project when the assumptions hold true.

### Limitations of the metric

Here, we applied the <*RMSD*_*cons*_> as our proxy for affinity. With respect to *RMSD*_*cons*_, for example, the linker may contribute, only a small subset of the conserved region could contribute, or other structural features may be more important, such as r-group torsional propensity or relative polar group vectors. Though *RMSD*_*cons*_ may offer a good naive metric, we recommend that designers use judgment and testing to choose the appropriate structural metric. Although the expectation value is a simple non-parametric measure of the distribution of the values, it may not be the ideal way to combine results from multiple conformers. An alternative metric could be, for example, the probability of the compound meeting an *RMSD*_*cons*_ threshold. This would make sense if the compound only binds if it meets that RMSD threshold (but binds no better or worse the otherwise larger or smaller the *RMSD*_*cons*_ gets). For example, if a 1.0 Å structure were necessary to bind, the function would be *P(RMSD*_*cons*_ <=*1.0 Å)*. A similar metric was applied by McCoull *et al*. in a BCL6 macrocycle design project^16^.

### Diagnosing the method

**Figure 5:**
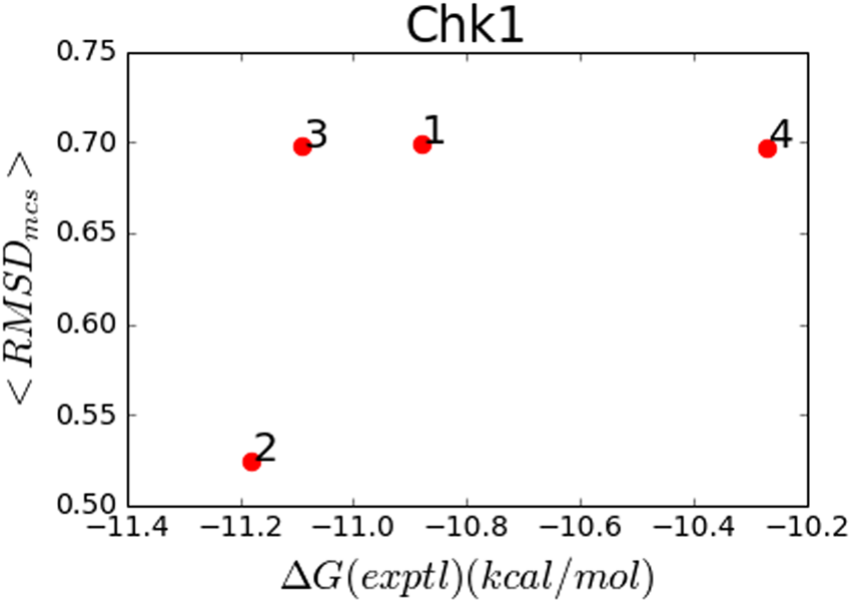
Proxy affinity metric, <*RMSD*_*cons*_> vs experimentally measured affinity for Chk1 with a smaller conserved region.

Due to the many assumptions of the method, it may not be clear *a priori* whether this method is applicable to the macrocycle design project at hand. It is recommended that the designers confirm that the strain-rmsd profiles (Fig. 3a–c) behave as expected, that the low energy conformations of any known macrocyclic binders can access the bioactive substructure. Limitations of the metric (as mentioned above), and sampling protocol may need to be adjusted. For example, if we were to use the Chk1 results as a retrospective to propose prospective linkers, the aberrant behavior of compounds 3 would draw further critique. This could possibly be explained by the large MCS region (visible in Fig. 2a) which extends through most of the linker. One could argue that though this region is “conserved” (at least by the MCS method), it is not a good representation of the bioactive substructure. Performing the calculation with an abbreviated SMARTS pattern to determine the conserved region, *n1c(O)cncc1NC(*=*O)Nc2c(O)ccc(c2)Cl*, which removes now omits most of the linker other than the first atom on each end, suggests improved results (R^2^ goes from from 0.05 to 0.27, though the p-value, 0.48, does not suggest this is statistically significant), see Fig. 5.

The sampling itself can potentially be an area where modification to the protocol is necessary. For example, for an extremely large macrocycle (>40 ring atoms), increasing sampling settings or constraints to focus the sampling may be necessary. Additionally, since the hypothesis relies on the impact of the cycle topology on the conformation, interactions of extended side chains, for example, may introduce noise into the strain calculation.

Additional concerns about proxy metric, force field, and solvent parameters may also be investigated by the modeller in a project-dependent manner. The default solvent and force field parameters were used here. We did also redo these calculations in VSGB2.1 solvent model^26^ to see if that would significantly affect the results. The results were approximately unchanged, yielding a weighted R^2^ of 0.61 (from 0.60). This change is insignificant and we do not believe the improvement is worth rationalization.

### Use as a screening tool

The assumptions of this method can be managed when evaluating a large number of cyclizations or otherwise enumerated macrocycle topologies for purpose of structural stabilization. For such cases, thousands of compounds can be quickly triaged down to those few compounds likely to maintain the bioactive conformation. This tool could potentially be coupled with automated design tools such as linker enumeration or genetic-algorithm-type linker evolution methods. However, beyond this, more accurate screening methods, such as free energy perturbation, will be necessary to further probe interactions, solvent effects, or more precisely determine entropic factors contributing to the free energy of binding.

## Conclusions

We have described a high-throughput method for profiling macrocycles propensity to adopt the bioactive conformation of their linear progenitor and shown that this propensity is a useful proxy for the binding affinity in cases where the binding mode is both constant and known and when the linker is not forming interactions with the protein. The systems here were limited to publically available data. But despite this limitation and the simplicity of the macrocycle stabilization algorithm, good performance was observed (R^2^ weighted average 0.60). We expect that in a design context, the datasets will be larger and the <*RMSD*_*cons*_> metric will likely have to be modified to properly test the macrocyclization strategy based on the particular structural motivation. For example, as demonstrated in the discussion section, a slight, rational modification significantly improved the correlation. We have also proposed options and rationales for varying the metric for such cases. We note that, though care must be taken to ensure that the results here are not over-extrapolated (e.g. since the metric cannot, in any way, predict new interactions or differential solvation effects), this method could be extremely useful in rapidly triaging large numbers macrocycle candidate compounds.
